# Settling aerodynamics is a driver of symmetry in deciduous tree leaves

**DOI:** 10.1098/rsif.2024.0654

**Published:** 2025-05-07

**Authors:** Matthew Dominic Biviano, Kaare Hartvig Jensen

**Affiliations:** ^1^ Technical University of Denmark, Lyngby, Hovedstaden, Denmark; ^2^ Department of Physics, Danmarks Tekniske Universitet, Lyngby, Denmark

**Keywords:** plant biomechanics, sedimentation, fluid mechanics, biomimetics, morphology

## Abstract

Leaves shed by deciduous trees contain 40% of the annually sequestered carbon and include nutrients vital to the expansion and health of forest ecosystems. To achieve this, leaves must fall quickly to land near the parent tree—otherwise, they are lost to the wind, like pollen or gliding seeds. However, the link between leaf shape and sedimentation speed remains unclear. To gauge the relative performance of extant leaves, we developed an automated sedimentation apparatus capable of performing approximately 100 free-fall experiments per day on biomimetic paper leaves. The majority of 25 representative leaves settle at rates similar to our control (a circular disc). Strikingly, the *Arabidopsis* mutant asymmetric leaves1 (*as1*) fell 15% slower than the wild-type. Applying the *as1-digital* mutation to deciduous tree leaves revealed a similar speed reduction. Data correlating shape and settling across a broad range of natural, mutated and artificial leaves support the *fast-leaf hypothesis*: deciduous leaves are symmetric and relatively unlobed partly because this maximizes their settling speed and concomitant nutrient retention.

## 1. Introduction

**Figure 1.**
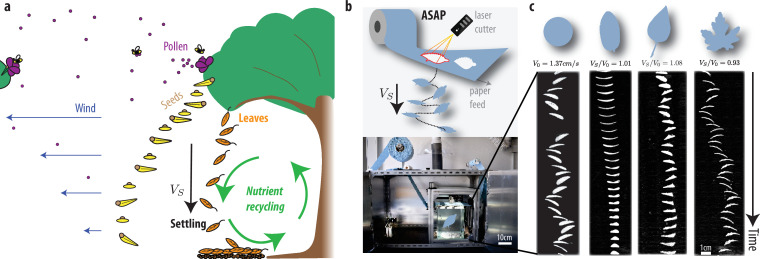
The fast-leaf hypothesis. (a) Trees shed pollen and seeds which are dispersed aided by the wind (and by animals). Leaves shed by deciduous trees include nutrients vital to the expansion and health of forest ecosystems. However, the connection between the sedimentary behaviour of the leaves and their shape remains unexplored. We propose that the fate of leaves serves the role of enriching the local environment thus evolving to symmetric shapes that maximize the settling speed $V_{S}$. As discussed by Viola & Nakayama [1], dispersion range is linearly related to wind speed. Note that while wind speeds are markedly lower in dense forest environments such as tropical rainforests [2], the relative advantage of faster settling symmetric leaves persists. (b) Schematic of the automated sedimentation apparatus and a photograph of the device. (c) Representative sedimentation data showing from left to right our control (a circular disc), *Amanchelier arborea*, *Syringa reticulata* and *Acer saccharinum*. See also the electronic supplementary material, video S1 and figure S10.

Deciduous trees annually shed many parts of themselves; including leaves, pollen, seeds and fruits (figure 1a). Seeds and pollen have evolved aerodynamic traits allowing them to be dispersed far and wide lassisted by the wind [3,4]. However, despite the ubiquity of falling leaves, it is unclear how their aerodynamic properties interact with the evolutionary story of the tree. In this work, we propose that symmetry in deciduous leaf shape has evolved through natural selection to enhance rapid settling. This trait probably facilitates local nutrient recycling through the soil thereby improving the competitive fitness of trees and their offspring.

Deciduous trees shed their leaves annually, losing approximately 40% of their assimilated carbon and also substantial quantities of nutrients (e.g. N, P, K, Mg and Ca; [5]). Evolutionary thinking suggests that trees may optimize their leaves to deposit where their nutrients can be recycled, and locally improve soil conditions [6]. Species that use limiting nutrients more efficiently should have a competitive advantage [7]. Natural selection has promoted distinct physical traits that enhance the dispersal of seeds, fruits and pollen, across a relatively broad geographical range [3]. Shape adaptations that take advantage of aerodynamic effects allow, e.g. the gliding Javan cucumber seed or the helicopter maple seeds to fall slowly and be carried by the wind to settle remarkably far from the parent plant [8,9]. Another notable example is the dandelion pappus, whose (three-dimensional) porous structure contributes to its slow descent owing to a large drag-inducing wake that stabilizes its fall and enables long-distance wind dispersal [1].

In contrast to pollen and seeds, however, leaves must fall to the ground quickly to achieve a proximal concentration of nutrients. Indeed, leaf removal from *Fagus sylvatica* groves was shown to remove 1.5 t ha^−1^ of carbon from the soil, corresponding to 30–70% of the vital nutrients with concomitant effects on growth [6]. Other potentially beneficial effects of leaf anti-dispersal include shading of competing species [10], mediating earthworm activity [11] and influencing moss growth via the soil chemical composition [12].

The starkest difference between leaf and seed pod aerodynamic design is the relatively high symmetry of leaves. The molecular origins of symmetry during leaf morphogenesis are well established (e.g. [13]); however, exploring the quantitative reasons *why* leaves are symmetric has attracted relatively little attention.

The evolutionary origins of extant leaf shape have been placed into the context of, e.g. developmental constraints, reconfiguration to minimize wind loads, gas exchange and temperature control (e.g. [2,4,14]). Biomechanical rationales for symmetric transport networks have also been proposed, including resilience to damage [15] and mechanical stability [16]. Several authors have advocated for symmetry as a basal state in leaves [17,18]. There is some experimental evidence to support this idea. For instance, some early multicellular organisms (e.g. red algae) comprised approximately symmetric cell clusters [19], and similar patterns have been described in early land plants [20]. However, several extant species have asymmetric leaves, for example, in genii such as *Eucalyptus* [21] and *Begonia* [22]. Similarly, bilateral symmetry is not unambiguous in the fossil record: asymmetry has been noted in early multicellular organisms such as green algae [23]. The structures of *Asteroxylon mackiei*, a lycophyte with microphyll leaves, described in the Rhynie chert further highlight the diversity of ancestral plant forms [24,25]. While these studies focus on shoot morphology and evolutionary transitions, they illustrate the complexity of early plant development and challenge the notion that symmetry is a universal ancestral trait.

The available evidence does not allow us to rule out the possibility that symmetry is not a basal trait. In particular, it remains unclear how leaf shape interacts with the settling and subsequent nutrient recycling processes. Here, we examine the idea that rapid settling associated with the leaf recycling process limits the diversity of leaf shapes. Exploring representative mutations to existing leaf morphologies, we demonstrate that rapid settling explains the ubiquity of symmetric blades. Our specific aims are to: (i) investigate how the symmetry and lobedness of deciduous leaves influence their settling dynamics; (ii) quantify the relationship between leaf shape and sedimentation speed using experimental data and digital mutations; and (iii) validate the fast-leaf hypothesis, which posits that symmetric and unlobed leaves settle faster, facilitating nutrient retention and local recycling.

While this study focuses on isolating aerodynamic factors through a simplified experimental framework, we acknowledge that evolutionary processes are influenced by a wide range of developmental, ecological and physiological factors. Our findings should, therefore be viewed as a mechanistic exploration of one aspect of leaf morphology.

## 2. Methods

Each leaf has its own unique shape and falls in a distinct way depending on factors such as growth patterns and atmospheric conditions [26]. Our experimental design aims to isolate the aerodynamic effects of leaf shape. While this provides a clean testbed for studying settling dynamics, it does not encompass the full complexity of natural environments or evolutionary pressures. To ensure accuracy and prevent any unwanted confounding effects, we developed a standardized experimental set-up to isolate leaf shape effects (figure 1b): The automated sedimentation apparatus (ASAP) enables us to examine the falling trajectory of settling paper leaves. Shapes cut by a laser from a ticker tape paper roll fall directly into our observation tank, where a camera records the leaf motion (figure 1c). This vertical configuration obviates the need for multi-axis robotic manipulation and vacuum lines in previous studies [27]. To isolate the shape as a variable, we maintained a constant leaf blade area A=100 mm${,}^{2}$. We observed no significant difference in the settling velocity when varying the leaf mimic’s absolute area (electronic supplementary material, figure S5) or initial orientation (electronic supplementary material, figure S6). For further information on the tracking software and set-up, please refer to the electronic supplementary material.

Leaves falling freely in air experience Reynolds numbers (Re) ranging from a few hundred to few thousand, while leaves fixed to the plant body can reach $Re=10^{4}$ in windy conditions [4]. Our experiment operated in the range $Re=v_{s}D/\nu \approx$100—300, where D≈1 cm is leaf radius, $v_{s}\approx 1$ cm s^−1^ is the typical settling speed, and $\nu =10^{-6}$ m s^−2^ is the viscosity of our operating fluid (water). Consistent with prior literature on drag of freely falling non-spherical particles [28], the relative performance different shapes in our experiment did not depend strongly on Re (electronic supplementary material, figure S9).

Like in a natural leaf settling experiment, the kinematics are highly dependent on initial conditions. Each shape trajectory was therefore recorded 20–30 times to determine the mean settling speed $V_{S}$ within 3% relative error. The relative settling speeds reported here are thus representative of the many thousands of leaves lost by each tree. By observing the relationship between leaf shape, velocity and falling behaviour across various shapes (figure 1c, electronic supplementary material, video S1), we can gain insights into aerodynamics, drag and leaf performance optimization.

## 3. Results

We investigated the sedimentation characteristics of 25 species from the families Ulmaceae, Styracaceae, Malvaceae, Sapindaceae, Rosaceae, Fagaceae, Bignoniaceae, Oleaceae, Ebenaceae, Eucommiaceae, Moraceae, Magnoliaceae, Betulaceae, Platanaceae and Salicaceae (electronic supplementary material, figure S1). This dataset comprised deciduous trees with simple leaves that grew to a sufficient height at maturity (> 5 m tall) and contained examples of major patterns that may affect the aerodynamic performance such as symmetry, lobedness, aspect ratio, toothedness and combinations of those parameters [29]. The annual herb *Arabidopsis thaliana* was also included because the *as1*-mutant presents asymmetry [13]. While biological variability in natural leaves was not directly included in this study, three leaves from each species were analysed.

To classify the shapes, we assess the leaves by their reflection symmetry [30] and their lobedness (electronic supplementary material, figure S2). The leaf symmetry number S quantifies reflection symmetries (*S* « 1 no symmetry, *S* = 1 one or more axes), while the lobedness number *L* quantifies the mass distribution (*L* « 1 unlobed, *L* = 1 highly lobed).

**Figure 2.**
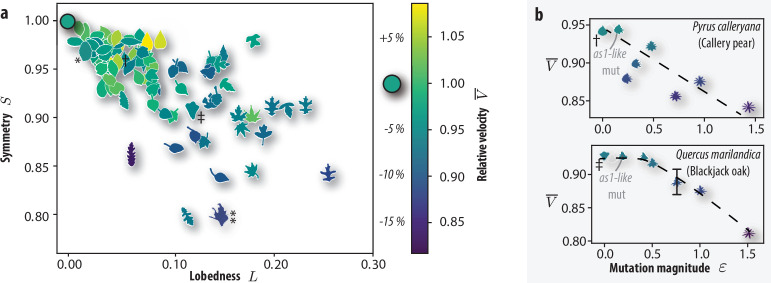
Exploring the interplay between leaf shape and terminal sedimentation speed using digital mutations. (a) Leaf morphology quantified by symmetry S and lobedness L (electronic supplementary material, figure S2) and their effect on terminal settling velocity. Three leaves from each species were analysed. The terminal velocity (colour bar) is scaled by the terminal velocity of the circular control disc. *Arabidopsis thaliana*, wild-type (*) and *as1* mutant leaves (**) are highlighted, as well as pear oak leaves which were subjected to digital mutations. (b) The effects of digital mutation on terminal velocity. Pear and oak leaves were progressively mutated by adding a short wavelength sinusoid to segments of their boundary. We quantify the digital mutation magnitude $\varepsilon =\Delta A/A_{0}$, where $A_{0}$ is the area of the original shape and ΔA is the area modification of the shape. An error bar is placed in the *Quercus marilandica* dataset to demonstrate the typical variance of the mean sedimentation velocity for that shape. Dashed lines are provided as a guide for the eye to highlight the main data trend: settling speed decreased with increasing mutation amplitude. The raw data underlying this figure, including settling velocities, symmetry and lobedness metrics, are provided in the accompanying supplemental dataset (see also the electronic supplementary material, figure S1; [28,31–36]).

Deciduous leaves tend to be symmetric (*S* = 0.85–1) and subtly lobed (*L* = 0–0.2), see figure 2. For instance, *Arabidopsis* is symmetric and unlobed (L,S)=(0.02,0.96), while some members of the *Quercus* and *Acer* families are lobed and somewhat asymmetric (L,S)=(0.2,0.9). Despite this variation, most extant leaf shapes fall at a velocity within ±10% of a circular disc of equal mass. Interestingly, *Arabidopsis as1* mutants leaves settle notably slower (−15%). In this mutant, organ patterning is disrupted and the leaf phenotype is asymmetric (figure 2) (L,S)=(0.14,0.80). This leads us to question whether a causal link between symmetry, lobedness and settling speed exists.

To tease out the effect of a leaf’s shape on its aerodynamics, we applied a shape modification to extant leaf outlines. Based on the aforementioned *as1*’s characteristic large amplitude/short wavelength perturbations, we create *digital mutations* by sinusoidally perturbing segments of the leaf edge. This allows us to gauge the relative performance of different phenotypes that are, in principle, immediately accessible to natural selection. We applied digital mutations to the bell-shaped blackjack oak (*Quercus marliandica*) and the ovular callery pear (*Pyrus calleryana*; figure 2b) leaves. As the mutation strength (modified area fraction ε) grew, a striking 15% drop in the terminal velocity was observed in both cases. The pear leaf responded noticably to even small/asymmetric pertubations, while the oak’s initial response was less pronounced.

**Figure 3.**
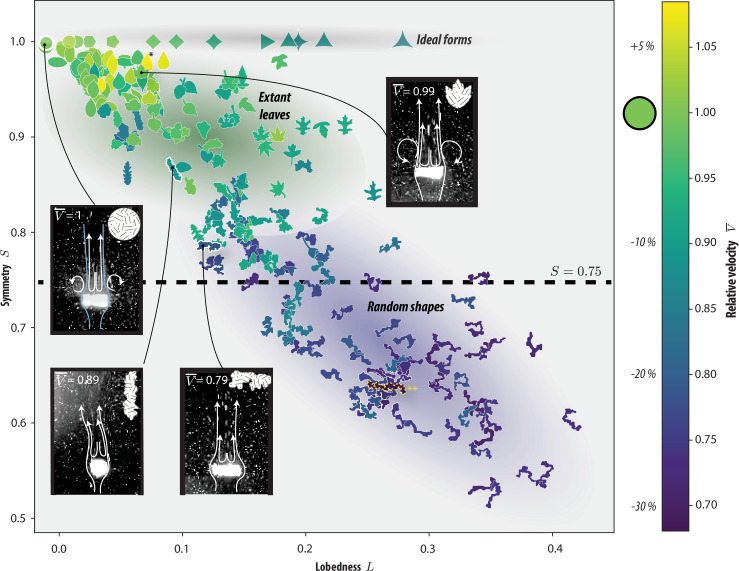
The effect of symmetry and lobedness on the terminal velocity. The terminal velocity of 25 extant leaves, 10 ideal forms and 120 shapes generated from random walk outlines (electronic supplementary material, figure S3) are compared. High-symmetry shapes (S>0.75, dashed horizontal line) settled at speeds on par with a circular disc (within ±10%), and overall the speed decreased with increasing lobedness in this regime. By contrast, the settling of asymmetric objects (S<0.75) was substantially slower: speeds systematically decreased −15 to −30% and were largely insensitive to the shape's lobedness. The fastest, *Syringa reticulata* (+10%, *), and slowest, a slender meandering form (−30%,**), are labelled for reference. To probe the fluid flow behaviours of various shapes, a laser sheet and 0.02 mm tracer particles dispersed in the solution were used to generate the inset images (see magnified view in the electronic supplementary material, figure S8). The raw data underlying this figure, including settling velocities, symmetry and lobedness metrics, are provided in the accompanying supplemental dataset.

Having explored the shape and settling characteristics of natural and mutated leaves, we can now formulate the *fast-leaf hypothesis*: natural leaves are highly symmetric and relatively unlobed because this maximizes their settling speed and the concomitant nutrient retention. The aforementioned data support this conjecture; however, important questions remain unanswered. Specifically, we inquire how plant leaf shape and sedimentation traits are positioned among all possible two-dimensional geometries? To further explore the impact of symmetry and lobes on sedimentation, we generated 120 random shapes from the outline of the path traced by a diffusing particle ([37]; figure 3; and elecronic supplementary material, figure S3). The set of two-dimensional forms now covered a relatively wide qualitative and quantitative range of morphologies: from regular polygons across leaves to random structures (*L* = 0—0.4 and *S* = 0.5—1).

Sedimentation experiments on this broad set of shapes revealed two regions, roughly separated by a symmetry value of S=0.75 (figure 3). High-symmetry shapes (S>0.75) settled at speeds on par with a circular disc (within ±10%), and overall the speed decreased with increasing lobedness in this regime. By contrast, the settling of asymmetric objects (S<0.75) was substantially slower: speeds systematically decreased by −15 to −30% and was largely insensitive to the shape’s lobedness. Generally speaking, asymmetric leaf mimics rotated slowly while falling steadily (electronic supplementary material, figure S7). We speculate that irregular morphologies reduced flow features often associated with fluttering and tumbling. Indeed, the stark reduction in speed from high to low symmetry also markedly changed the flow near falling objects. Particle tracking (figure 3, inserts) revealed that fast shapes have long wakes with strong external recirculating and often transitioned to pitching behaviour, consistent with Ern *et al*. [38] and Willmarth *et al*. [39]. By contrast, slower shapes had shorter wakes and only recirculation within the main wake. This effect is illustrated for natural and mutated *Amanchelier arborea* leaves in the electronic supplementary material, video S1.

From these quantitative and qualitative data, it is clear that symmetry, and to a lesser extent lobedness, is a strong predictor of high settling velocities. This lends additional support to the fast-leaf hypothesis: symmetric forms settle fast, and no substantially faster shapes available to natural selection were identified. We acknowledge, however, that fitter shapes may exist among forms not based on random walk outlines [37], and that porosity (not considered here) could be an important flow-control parameter [8,40].

## 4. Discussion and conclusion

Our data demonstrate that settling aerodynamics strongly limits leaf morphology. In the context of our research aims (§1), we have found that: (i) symmetry and subtle lobedness are predictors of high settling velocities in deciduous leaves; (ii) digital mutations that increase asymmetry or lobedness lead to significant reductions in settling speed; and (iii) the fast-leaf hypothesis is supported by our data, highlighting the evolutionary advantage of symmetric leaf shapes for rapid settling and nutrient retention.

Our results provide a mechanistic understanding of how aerodynamic performance might constrain leaf shape. However, we caution that these findings represent one component of a multifaceted evolutionary process. Factors such as habitat, climate and developmental genetics also play critical roles in shaping leaf morphology, and further studies integrating these dimensions are essential to build a more comprehensive picture. In particular, we acknowledge that our standardized approach removes phenotypic variability within species. However, this standardization allows us to isolate the effects of shape on settling dynamics. Future work could expand on this by incorporating natural leaf samples to investigate the interplay between phenotypic variability and aerodynamic performance.

Data from wild-type and *as1*-mutated leaves suggest that high symmetry is a critical factor in rapid settling (figure 2b). A comprehensive comparison to random forms confirms this picture and suggests that extant leaves are near-optimal (figure 3), in the sense that their symmetric and minimally lobed shapes maximize settling speed and aerodynamic stability, facilitating nutrient recycling and local deposition. This suggests that their morphology has evolved to balance aerodynamic performance with ecological function. At present, realistic shape mutants provide extant trees with minimal access to increased fitness (i.e. faster settling speed) by natural selection. However, mutated leaves that will occur owing to climate change [26,41,42] are generally inferior. This points towards a new positive feedback mechanism for atmospheric carbon.

It is worthwhile to consider the limitations of this study in the context of non-deciduous plants. An important feature of non-deciduous leaves is that they are significantly denser than deciduous leaves. For instance, Poorter *et al*. [43] found that the leaf mass per area is up to 10 times larger (mean 2.5 times). This, of course, changes the settling dynamics because heavy objects are relatively less affected by drag and thus settle faster [44]. While the relatively high density of non-deciduous leaves may have evolved primarily to mitigate effects of, for instance, drought, cold and herbivory, nutrient recycling via rapid settling is another advantage. To compare with non-deciduous plants, we conducted settling experiments on leaf mimics variable area density, confirming previous studies of the positive correlation between speed and area density (electronic supplementary material, figure S4).

In this study, we focused on isolating the effects of the blade shape on settling rates, and the petioles were removed from the analysed leaves to standardize the shapes. Including petioles could provide additional insights into the dynamics of leaf sedimentation. Petioles, being along the axis of symmetry, might alter the centre of mass and potentially dampen or enhance rotational instabilities during settling.

In dense forest environments, wind speeds are significantly reduced compared with open settings [2]. Despite this, the relative difference in settling dynamics between symmetric and asymmetric leaves remains, preserving the evolutionary advantage of rapid settling for nutrient recycling. Moreover, as described by [1], the relationship between wind speed and dispersion is linear, meaning even low-wind conditions can contribute to measurable dispersion. While our study isolates the settling dynamics of leaves under still-air conditions, natural leaf shedding often occurs in the presence of wind, which can influence both the detachment process and subsequent dispersal. Studies by Spicer [45] and Greenwood & Donovan [46] have examined the transport and deposition of plant material under various environmental conditions, highlighting the interplay between leaf morphology and wind dynamics. Furthermore, Burnham [47] explored how these factors contribute to the sorting and preservation of plant macrofossils. Future work could extend our findings by incorporating wind-tunnel studies or field experiments to bridge the gap between controlled and natural environments.

With a renewed focus on the carbon cycle and forest health, it is more critical than ever to understand how carbon is managed by trees. The connection between leaf symmetry and the fate of leaves in forests and other ecosystems provides a simple, quantifiable link between the health of the ecosystem and morphology of leaves. Climate change has been shown to heavily modulate tree leaf shapes [26,41] and can impact the symmetry of leaves [42]. The link between the canopy and the soil may also be a major factor in stopping outdated practices such as leaf litter harvesting [6,48] that are still prevalent today. Moreover, patterns in the fossil record may have been influenced by leaf sedimentation [49]. Finally, we speculate that real and synthetic leaves may be a new and fruitful source of inspiration for aeronautics.

## Data Availability

Raw data available in the electronic supplementary material [50].
